# Investigation on the insecticidal activities of cyanobacterial extracts as an alternative source for the management of fall armyworm, *Spodoptera frugiperda* (J. E. Smith) (Lepidoptera: Noctuidae)

**DOI:** 10.1016/j.heliyon.2024.e29060

**Published:** 2024-04-03

**Authors:** Sharanappa C.H., Bheemanna M, Prabhuraj A, Harischandra R. Naik, Nagaraj M. Naik, Saroja N. Rao, Ihab Mohamed Moussa, Roua A. Alsubki, Fazal Ullah, Hosam O. Elansary, Kariyanna B

**Affiliations:** a Pesticide Residue and Food Quality Analysis Laboratory, University of Agricultural Sciences, Raichur, Karnataka, India; b School of Agricultural Sciences, Malla Reddy University, Hyderabad, India; c Department of Botany and Microbiology, College of Science, King Saud University, P.O. Box 2455, Riyadh, 11451, Saudi Arabia; d Department of Clinical Laboratory Science, College of Applied Medical Science, King Saud University, Saudi Arabia; e State Key Laboratory of Grassland Agroecosystems, College of Ecology, Lanzhou University, Lanzhou, 730000, China; f Plant Production Department, College of Food and Agriculture Sciences, King Saud University, Riyadh, 11451, Saudi Arabia; g Fluoro-Agrochemicals, CSIR-Indian Institute of Chemical Technology, Tarnaka, Hyderabad, India

**Keywords:** Cyanobacterial, Fall armyworm, *Nostoc muscorum*, *Spirulina* sp. LC_50_, GC-MS

## Abstract

The *Spodoptera frugiperda* is a notorious pest with a broad host range. It severely damages crops, mainly in areas of the globewhere maize and sorghum are grown. The pest is difficult to control due to its adaptive nature and resistance to several insecticides available in the market. So, an identification of the alternative strategy is the prime important in the present context. Insecticidal activities of cyanobacterial extracts were evaluated in the laboratory as a biocomponent against *S. frugiperda*. The crude extracts of *Nostoc muscorum* and *Spirulina* sp. were prepared by using ethanol, methanol and petroleum ether solvents. Soxhlet apparatus was used for extraction. *S. frugiperda* larvae in their second instar were given access to fragments of maize leaf that had been treated with various cyanobacterial extracts. The findings displayed that the petroleum ether extract of *N. muscorum* had the lowest LC50 value of 155.22 ppm, followed by petroleum ether extracts of *Spirulina*, ethanol extract of *N. Muscorum,* methanol extract of *N. muscorum*, ethanol and methanol extract of *Spirulina* with an LC_50_ values of 456.02, 710, 780, 1050 and 1070 ppm respectively. Later, the effect of LC_50_ values on many biological parameters like the larval duration and pupal stages, the percentage of pupation, the weight of the pupal stage, the malformation of the pupal and adult stages, adult emergence percentage, fertility and the longevity of the male and female adult stages of *S. frugiperda* was examined. The gas chromatography-mass spectrometry (GC-MS) was used to analyse the crude extract to identify the bioactive components that were responsible for the insecticidal properties. The major compounds detected were diethyl phthalate (19.87 %), tetradecane (5.03%), hexadecanoic acid, ethyl ester (4.10 %), dodecane (4.03%), octadecane (3.72%), octadecanoic acid, methyl ester (3.40 %), ethyl oleate (3.11 %), methyl ester. octadecenoic acid (3.04 %), heptadecane (3.04 %) and phytol (3.02 %). The presence of several bioactive chemicals in the cyanobacterial extracts may be the reason for their insecticidal actions, thus it can be used as an alternative and new source to combat fall armyworm and other crop pests.

## 1 Introduction

Worldwide, environmental contamination is a major problem as a result of the increasing rate of urbanization and industrialization. Specifically, the increased application of chemical pesticides has leads to numerous environmental and ecological problems in various countries [1]. One of the objectives of scientific study is the demand for natural insecticides. The term "biopesticides" refers to a significant class of naturally derived, slow-acting crop protection agents which are less hazardous to human and environment compared to synthetic chemicals. Cyanobacteria are photosynthetic prokaryotes and morphologically varied groupings that thrive in a variety of environments. Cyanobacteria exhibit amazing diversity in terms of morphology, ranging from filamentous to unicellular and colonial forms. A significant number of cyanobacteria species are found in terrestrial, marine or freshwater environments, according to Cameron [2]. Cyanobacteria have been found to produce a varieties of metabolites with numerous bioactivities [3].

*Spirulina* is a type of freshwater spiral prokaryote with a single cell. It is a gram-negative, filamentous cyanobacterium and non-nitrogen-fixing photoautotrophic bacterium. It includes the essential minerals, vitamins A, B, E, and K and fatty acids that the body needs, as well as protein components (60–70%) made up of 18 different amino acids [4]. Because of its high protein, vitamin, and mineral content, spirulina is considered a nutrient-dense diet [5]. *Spirulina* can also help to treat diabetes, anemia, cancer, cardiovascular disease and arthritis [6]. According to Supramaniyan and Jeeji [7], *Spirulina* grows best at temperatures between 25 and 30 °C and pH levels of 8 and above. Although *Spirulina* is good for humans, *S. platensis* water and ethanolic extract increased the mortality of larvae and affected several biological parameters of *Spodoptera littoralis* [8,9]. A free-living microbe called *Nostoc muscorum* (cyanobacteria) can be found in both freshwater and terrestrial habitats [2]. Gram-negative, filamentous, greenish-brown *N. muscorum* have the ability to generate spores when dried. *N. muscorum* is essential for the fixation of the carbon and nitrogen in soil environments. *N. muscorum* thrives best in environments with pH levels between 7.0 and 8.5, with a lower pH limit of 5.7 as well as light levels are lower than those of bright sunshine. Despite the lack of sunshine and the presence of glucose, it can still grow and fix nitrogen [10].

The metabolites were probably responsible for regulating the community since they first appeared in cyanobacterial mats. Production of these metabolites is largely species as well as strain-dependent [11]. According to Katrikioglu et al. (2006), extracellular and intracellular metabolites with a range of biological activities, including antiviral, antibacterial, and antialgal properties were produced by different strains of cyanobacteria. Algal species and the solvents used to extract those both affect antimicrobial activity [12,13]. Many secondary metabolites with a range of bioactivities are produced by cyanobacteria [14,15].

A number of studies on algal and cyanobacterial extracts that scientists reported as insecticides included, *Dolichospermum flos-aquae*, (Bornet & Flahault) Hrouzek & Ventura*, Anabanea fertilissima*, *Anabanea laxa* and *Nostoc muscorum* were utilized as biopesticides against the *Agrotis ipsilon* (Hufnagel) [16]. Brown macro alga *Fucus vesiculosus* and blue-green microalga, *Arthrospira platensis* were reported as biocontrol agents against *Bruchidius incarnates* (Boheman) [17]. *S*. *frugiperda*, usually called as the fall armyworm (FAW), is a devastating insect pest belonging to the Noctuidae family within the Lepidoptera order. Long-term effects on food security are prompted by this polyphagous insect, which damages crops *viz.,* maize, sorghum, rice, cotton, and other vegetable which are economically important [18,19]. The first report on maize in India was noticed in Shivamogga, Karnataka, in 2018 [20]. FAW attacks the stems, reproductive organs and leaves of various plant species. The pest is native to the subtropical and tropical regions of the Americas. It was initially emerged in America and a common pest of maize in both South and North America [21].

After being found in Africa for the first time in 2016, it had spread to over 30 southern and tropical African nations by the end of 2017 [22,23], including Madagascar, Seychelles, and Cabo Verde. It had expanded to more than 44 nations by the end of 2019 [24]. 353 plants have been reported to be affected by this pest, according to Kansiime et al. [25]. FAW severity has been associated with a wide host range, robust dispersal capacity, high fecundity rate, and lack of diapause [26]. Prior to oviposition, the pest can travel over 500 km [27]. Chemical pesticides have been widely utilized to increase crop yields and shield crops from insect pest damage. The necessity of natural pesticides is important goals of scientific research [16]. Pesticides derived from botanicals are a more environmentally friendly option for crop protection than chemical pesticides [28].

Thus, exploring natural substitutes for pesticides, the search for novel pesticides provide an opportunity to discover previously undiscovered chemical compounds. While the most of the global biomass comes from blue-green algae or cyanobacteria, which is a mostly unexplored source, plants do hold great potential for the development of novel pesticides. It has not been studied how well extracts of *Spirulina* sp. and *Nostoc muscorum* work as insecticides against *S. frugiperda*. In view of this, an investigation was conducted into the insecticidal properties of cyanobacterial extracts as a potential substitute source for controlling *S. frugiperda*.

## 2 Materials and methods

### 2.1 Cyanobacterial source

The strains of *Spirulina* sp*.* (NCIM, 5143) and *Nostoc muscorum* (NCIM, 2788) were collected from the National Centre for Industrial Microorganisms (NCIM), Pune, Maharashtra.

### *2.2* *Spirulina* sp. and *Nostoc muscorum* mass production media

For the mass production of *Spirulina* sp., Zarrouk media [29] and *Spirulina* media (NCIM, Pune) were utilized, while BG-11 media and fogs media (NCIM, Pune) [30] were used for *N. muscorum*.

### 2.3 Requirements for the growth and development of *Nostoc muscorum* and *Spirulina* sp.

A loopful of pure culture consist of 10 ml of liquid media was first added to test tubes for the purpose of subculturing them in a laminar airflow. After that, the test tubes were sealed with cotton wool and kept in a growth chamber with a steady illumination of light source at 25.2 °C and 75% RH. After a month, the inoculation cultures had reached their full growth capacity. Then, 100 ml of broth and 1 ml of the culture were mixed together, were subsequently placed in a growth chamber (Rahim and Hamed, 2013) with a little modification. To get an adequate volume of culture, the same procedures were used for 500, 1000, 2000, and 3000 ml of broth. The culture needs to be maintained in different amounts of liquid media because of its low dry weight.

### 2.4 Harvesting biomass from *N. muscorum* and *Spirulina* sp. species and converting it into a powder

The fully formed culture was allowed to air dry for a week at room temperature in a shady area after being filtered through muslin cloth. An electric blender was used to grind up the cultures. *N. muscorum* and *Spirulina* sp. in dry powder form were preserved in a refrigerator (4 °C) till further use [31].

### *2.5* *N. muscorum* and *Spirulina* sp. extraction

For the extraction, 5 g of *N. muscorum* and *Spirulina* sp. powder were utilized. For extraction by using ethanol, petroleum ether (PE) and methanol, Soxhlet apparatus were used.

### 2.6 Automated soxhlet extraction

Using automated soxhlet apparatus, 5 g of the powdered substance were extracted with 120 mL of ethanol, petroleum ether and methanol. Desiccator was used to obtain the crude extracts concentration method. Crude extracts were dissolved in dimethyl sulfoxide and stored at 4 °C to make standard stock solutions. Extractions were done in line with Rahim and Mohammed (2013), but a little different.

### 2.7 Maintenance of insect cul

A circular insect breeding dish with chopped maize leaves inside was used to raise the larvae. The dish was covered and kept at 25 °C, L12:D12 photoperiod and 75°RH. The adults that had just emerged were permitted to lay eggs in cages. Each cage contains pairs of adult males and females. Paper towels were used as the oviposition substrate inside the cages. The adults in the cages were given a 10% honey solution to eat, which was soaked on cotton pads and presented in little plastic caps that were changed every day. After oviposition, eggs were gathered and incubated in a circular dish used for insect breeding. The eggs were checked every 12 h to see if they had hatched. For the bioactivity test, second larval instars were used (Sharanabasappa et al., 2018b).

### 2.8 Bioassay test

To evaluate the insecticidal activity of cyanobacterial extracts against *S. frugiperda*, the leaf dip method of bioassay was used. Six different concentrations were prepared from the stock solution containing 0, 100, 200, 400, 800, and 1600 ppm. The doses or concentrations were set using the bracketing method or the ad hoc method. The chopped pieces of maize leaf were used to feed *S. frugiperda* second instars after they were dipped in extracts for 30 s and then allowed to air dry at room temperature [32,33]. The larvae were fed fresh maize leaves until they pupated, which occurred after 24 h of feeding on treated leaves [34]. Each concentration was tested three replications (20 larvae/replication), daily changing the dried leaves with fresh, untreated leaves. LC50 values were determined 96 h later.

### 2.9 Effect of *N*. *muscorum* and *Spirulina* sp. extracts at median lethal concentrations on different biological parameters

It was noted that the LC_50_ values of *N. muscorum* and *Spirulina* sp. extracts had a harmful effect on a number of biological parameters, including length of pupal weight, larval and pupal duration and the percentage of pupation, pupal and adult malformation, adult emergence, male and female adult longevity.

### 2.10 Statistical analysis

The LC50 values of the test results were calculated and corrected using the Abbott formula following 96 h of treatment [35]. Probit analysis [36] was used to evaluate the data, and POLO plus (Leora software 2002, Brkeliy, CA, USA) was used to analyse the lethal concentration (LC50) and associated 95 percent confidence limits (CLs). ANOVA (analysis of variance) along with Duncan test, op stat, and Wasp 2 software, were used for bioassay data on various biological parameters of *S. frugiperda*. The values have undergone transformations to square root and arc sine wherever needed.

## 3 Results

Larvae of *S. frugiperda* during the second instar were fed maize leaves treated with various cyanobacterial extracts, it was shown that the *N. muscorum* petroleum ether extract had the lowest LC50 value, measuring 155.22 ppm. Then came *N. muscorum* ethanol extract (710 ppm), *N. muscorum* methanol extract (780 ppm), and *Spirulina* petroleum ether extract (456.02 ppm). Conversely, higher LC50 values of 1050 and 1070 ppm, respectively, were recorded by the *Spirulina* ethanol and methanol extracts (Table 1).

**Table 1:** Toxic effect of *Spirulina* sp. and *N. muscorum* extracts against, *S. frugiperda*.

Treatment details	Leaf dip method				
	LC_50_ values (ppm)	Slope function (±SD)	Chi-square (df: 3)	95 % confidence limit	
				Upper	Lower
*Spirulina* PE ether extract	456.02	0.784 (±0.258)	0.043	1145.393	231.715
*N. muscorum* PE ether extract	155.22	0.755 (±0.242)	0.481	317.225	23.911
*Spirulina* ethanol extract	1050	0.982 (±0.241)	0.093	0.196	0.029
*Spirulina* methanol extract	1070	0.813 (±0.238)	0.064	0.388	0.088
*N. muscorum* ethanol extract	710	1.039 (±0.254)	0.067	0.121	0.024
*N. muscorum* methanol extract	780	0.937 (±0.248)	0.076	0.138	0.023

**ppm=** Parts per million **SD=** Standard Deviation **Df=** degrees of freedom.

### 3.1 The leaf dip method of bioassay was used to examine the toxic effects of *Spirulina* sp. and *N. muscorum* extracts at median lethal concentrations on various biological parameters of *S. frugiperda*

In comparison to their control, *S. frugiperda* larvae fed on maize leaf discs treated with various cyanobacterial extracts at their LC50 level significantly altered a variety of biological parameters.

#### 3.1.1 Larval duration

**Fig. 1:**
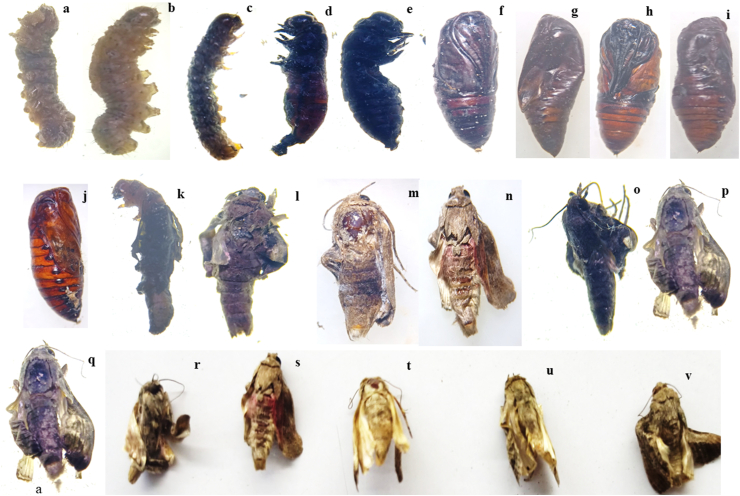
Effect of various cyanobacterial extracts on different biological parameters of *S. frugiperda*; a, b & c) larval malformations; d & e) larval-pupal malformations; f, g, h, i & j) pupal malformation; k & l) pupal-adult malformations and m to v) adult malformations.

The mean larval duration of second instar larvae of *S. frugiperda* fed on maize leaves treated with petroleum ether extract of *N. muscorum* at respective LC50 values increased significantly to 17.25 days. That followed by petroleum ether extract of *Spirulina* (16.67 days). The treatments, namely methanol and ethanol extract of *N. Muscorum* and methanol and ethanol extract of *Spirulina*, exhibited moderate effects and had mean times of 16.50, 16.33, 16.17, and 16.08 days, respectively. However, these treatments differed significantly from the control group, which had a mean of 15.42 days (Table 2). In addition, observed larval abnormality, figures (1a, b, and c) and (1d & e) show the larval-pupal malformation.

**Table 2:** Effect of cyanobacterial extracts on different larval and pupal parameters of *S. frugiperda*.

Treatments	Larval duration (days)	Pupal duration (days)	Pupation (%)	Pupal weight (g)	Pupal malformation (%)
*Spirulina* PE extract	16.67 (4.14)^b^	11.67 (3.49)^bcd^	59.20 (50.29)^abcd^	0.20 (0.84)^d^	45.83 (42.60)^cd^
*N. muscorum* PE extract	17.25 (4.21)^ab^	12.50 (3.60)^ab^	55.80 (48.36)^ab^	0.16 (0.81)^b^	52.50 (46.43)^ab^
*Spirulina* ethanol extract	16.17 (4.08)^bc^	10.92 (3.38)^cde^	64.20 (53.25)^cd^	0.24 (0.86)^f^	37.17 (37.56^)ef^
*Spirulina* methanol extract	16.08 (4.07)^bc^	10.67 (3.34)^de^	65.80 (54.25)^d^	0.25 (0.86)^f^	35.83 (36.77)^g^
*N. muscorum* ethanol extract	16.50 (4.12)^bc^	11.50 (3.46)^bcd^	60.80 (51.26)^abcd^	0.21 (0.84)^e^	41.50 (40.10)^de^
*N. muscorum* methanol extract	16.33 (4.10)^bc^	11.17 (3.41)^bcde^	62.50 (52.25)^bcd^	0.22 (0.85)^e^	39.17 (38.74)^ef^
Control	15.42 (3.99)^d^	10.00 (3.24)^f^	100 (90.00)^e^	0.29 (0.89)^g^	0.00 (0.00)^h^
**S. Em (±)**	**0.043**	**0.063**	**1.318**	**0.002**	**0.971**
**F value**	**1691.680**	**563.253**	**543.826**	**9280.633**	**435.937**
**CD (0.01)**	**0.146**	**0.276**	**4.302**	**0.013**	**3.772**
**CD (0.05)**	**0.106**	**0.201**	**3.122**	**0.009**	**2.738**

#### 3.1.2 Pupal duration

Petroleum ether extract of *N. muscorum*, at its LC50 values, significantly increased the pupal duration among the various treatments, with a mean of 12.50 days. Petroleum ether extract of *Spirulina*, ethanol and methanol extracts of *N. muscorum* were followed, all of which were at par with one another with a mean of 11.67, 11.50 and 11.17 days respectively and demonstrated a significant difference over the remaining treatments. *Spirulina* ethanol extract (10.92 days) and methanol extract of *Spirulina* (10.67 days) were the least effective in lengthening pupal duration, although they were still noticeably better than the control (10 days) treatments (Table 2).

#### 3.1.3 Percent pupation

With regard to the percentage of pupation, the petroleum ether extract of *N. muscorum* was found to be significant in reducing the percentage by 55.80%, followed by the petroleum ether extract of *Spirulina*, the ethanol extract and methanol extract *N. muscorum*, which were significant in reducing the percentage by 59.20, 60.80, and 62.50 percent, respectively. The methanol and ethanol extract of *Spirulina* on the other hand, were considerably better than control (100%) which was the recorded greatest percent pupation, but they were least efficient in lowering the percent pupation by 64.20 and 65.80 percent (Table 2).

#### 3.1.4 Pupal weight

The pupal weight of *S. frugiperda* was significantly lower, with a mean of 0.16 g, when they were fed on maize leaf discs treated with petroleum ether extract of *N. muscorum* at their LC_50_ value. The results of the ether extract of *Spirulina* petroleum (0.20 g), *N. muscorum* ethanol extract (0.21 g), and *N. muscorum* methanol extract (0.22 g), which were all considerably better than the other treatments and on par with one another. Ethonol extract of *Spirulina* (0.24 g) and methanol extract of *Spirulina* (0.25 g) were the least efficient in reducing pupal weight, although they were still noticeably better than the control (0.29 g), which had the highest pupal weight on record (Table 2).

#### 3.1.5 Pupal malformation

*N. muscorum* petroleum ether extract caused a mean of 52.50 percent pupal malformation, which was significantly higher than that of petroleum ether extract of *Spirulina* (45.83 percent). The ethanol (41.50 percent), methanol (39.17 percent) extract of *N. muscorum*, ethanol (37.17 percent) and methanol (35.83 percent) extract of *Spirulina* were comparable to each other but significantly better than the control, which had no malformation of pupae (0 %) (Table 2). Additionally, malformations of the pupae and pupa-adult were noted and depicted in figures (1f, g, h, I, j and k).

#### 3.1.6 Adult malformation

Among the various treatments, the adult deformity was considerably elevated by 43.67 percent when petroleum ether extract of *N. muscorum* was used at its LC_50_ value. Subsequently, *Spirulina* petroleum ether extract accounted for 33% of the total. The treatments like ethanol and methanol extract of *N. muscorum* and ethanol extract of *Spirulina* demonstrated significant superiority over the remaining treatments, with respective percentages of 31, 29.17, and 29.17*. Spirulina* methanol extract, on the other hand, was substantially better than the control, which had no influence on adult malformation (0%) but was least successful at causing adult malformation (26%). Adult malformations were displayed in Fig. 1 (l - v).

#### 3.1.7 Per cent adult emergence

When petroleum ether extract of *N. muscorum* was treated to maize leaves and offered to *S. frugiperda* second instar larvae, it considerably decreased adult emergence by 55.83 percent. These were followed by petroleum ether extract of *Spirulina* and ethanol extract of *N. muscorum*, which were on par with each other and significantly different over the other treatments by recording 57.17 and 59 percent, respectively. The treatments such as methanol extract of *N. muscorum*, ethanol and methanol extract of *Spirulina* were least effective in reducing adult emergence percentage by 61.83, 62.17, and 63.83 percent respectively, but significantly superior than the control that displayed the highest percent of adult emergence (Table 3).

**Table 3:** Effect of cyanobacterial extracts on different adult parameters of *S. frugiperda*.

Treatments	Adult malformation (%)	Adult emergence (%)	Fecundity (number)	Male adult longevity (days)	Female adult longevity (days)
*Spirulina* PE extract	33.00 (35.06)^d^	57.17 (49.13)^abc^	650.17 (25.45)^bcd^	6.42 (2.63)^b^	8.33 (2.97)^bcd^
*N. muscorum* PE extract	43.67 (41.36)^b^	55.83 (48.35)^ab^	606.00 (24.55)^ab^	6.13 (2.57)^ab^	7.92 (2.90)^ab^
*Spirulina* ethanol extract	29.17 (32.68)^ef^	62.17 (52.05)^bc^	738.67 (27.18)^cd^	6.92 (2.72)^bc^	8.83 (3.05)^cd^
*Spirulina* methanol extract	26.00 (30.64)^g^	63.83 (53.03)^c^	753.67 (27.46)^e^	7.08 (2.75)^c^	9.17 (3.11)^d^
*N. muscorum* ethanol extract	31.00 (33.83)^de^	59.00 (50.19)^abc^	725.17 (26.93)^cd^	6.58 (2.66)^b^	8.48 (3.00)^bcd^
*N. muscorum* methanol extract	29.17 (32.69)^ef^	61.83 (51.85)^bc^	728.33 (26.99)^cd^	6.75 (2.69)^b^	8.67 (3.03)^bcd^
Control	0.00 (0.00)^h^	99.50 (86.74)^d^	1064.83 (32.64)^f^	8.00 (2.91)^d^	10.50 (3.32)^e^
**S. Em (±)**	**0.692**	**1.247**	**0.743**	**0.071**	**0.042**
**F value**	**732.406**	**441.358**	**183.786**	**244.664**	**630.715**
**CD (0.01)**	**2.448**	**4.681**	**3.029**	**0.253**	**0.179**
**CD (0.05)**	**1.776**	**3.397**	**2.198**	**0.184**	**0.130**

#### 3.1.8 Fecundity

The reproduction rate of *S. frugiperda* was considerably reduced by all treatments using cyanobacterial extract or blue-green algal extract at respective LC_50_ values. Of these, *N. muscorum* petroleum ether extract displayed the lowest number of eggs, with a mean of 606. The next best treatments were petroleum ether extract of *Spirulina* (650.17 eggs), ethanol extract of *N. muscorum* (725.17 eggs), methanol extract of *N. muscorum* (728.33 eggs), and all of which were comparable to one another. *Spirulina* ethanol extract (738.67 eggs) and methanol extract (753.67 eggs) were shown to be less efficient in reducing the female egg layings, but they were still significantly better than the control group (764.83 eggs) (Table 3).

#### 3.1.9 Male adult longevity

The petroleum ether extract of *N. muscorum* drastically shortened the average male adult lifespan, which was 6.13 days, followed by ether extract of *Spirulina* petroleum had a mean of 6.42 days. The treatments like ethanol and methanol extracts of *N. muscorum* and ethanol extract of *Spirulina* were the next best treatments, recording 6.58, 6.75, and 6.92 days, respectively. *Spirulina* methanol extract, on the other hand, was considerably better than the control group (8 days), which had the maximum recorded adult lifespan, although it was still the least effective in decreasing the male adult longevity by 7.08 days (Table 3).

#### 3.1.10 Female adult longevity

Similarly, the mean lifespan of adult females was severely shortened by petroleum ether extract of *N. muscorum* at respective LC_50_ values, resulting in a mean of 7.92 days. These were followed by the following: petroleum ether extract of *Spirulina*, ethanol extract of *N. muscorum*, and *Spirulina* ethanol extract; their respective times of 8.33, 8.48, 8.67, and 8.83 days were comparable to one another. *Spirulina* methanol extract, on the other hand, was considerably better than the control group, which recorded the maximum female adult lifespan of 10.5 days, but it was least efficient in reducing female adult longevity by 9.17 days (Table 3).

### 3.2 Gas chromatography-mass spectrometry (GC-MS) analysis of cyanobacterial extracts to identify bioactive compounds against fall armyworm, *S. frugiperda*

According to the current study, *Spirulina* and *N. muscorum* extracts were prepared using organic solvents in order to identify bioactive components using GC-MS.

#### 3.2.1 Compounds detected in petroleum ether extract of *N. muscorum*

**Fig. 2:**
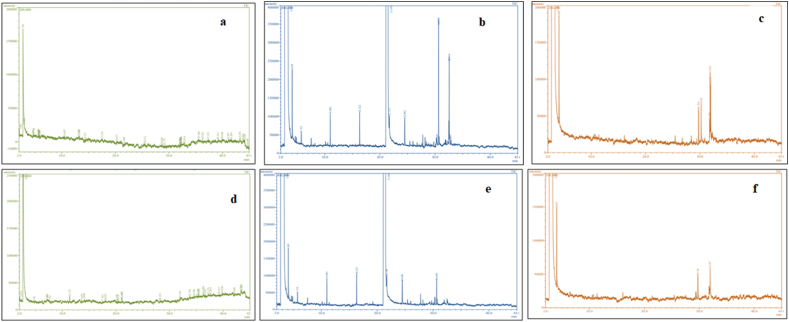
Gas chromatogram of the a) *N.muscorum* petroleum ether extract; b) *N.muscorum* ethanol extract; c) *N.muscorum* methanol extract; d)*Spirulina* petroleum ether extract; e) *Spirulina* ethanol extract; f)*Spirulina* methanol extract.

The petroleum ether extract of *N. muscorum* detected 35 compounds through GC-MS analysis. The compounds were mentioned according to their retention time in Table 4. The major compound detected were xanthine, 1-methyl-7-isopropyl-8-bromo (3.34 %), 10-methyl-3, 4, 5, 8, 9, 10-hexahydrooxecin-2-one (2.33 %), 8-hydroxy-2,2-dimethyl-8-phenyl-oct-5-en-3-one (1.88 %), bycyclo (2, 2, 1) heptanes-1-carboxylic acid (2.14 %), corynan-17-ol, 18, 19-didehydro-10-methoxy-acetate (ester) (1.86 %), *trans*-1,2-Diethoxycyclohexane (1.79 %), 4-heptenal, 6-(acetyloxy)-4-methyl-(E) (1.69 %), 4,8-dimethyl-10-oxo-methyl ester, undecanoic acid (1.68 %), docosanedoic acid, dimethyl ester (1.66%), octadecanoic acid, 16-oxo-, methyl ester (1.68 %), oleic acid and 3-(octadecyloxy) propyl ester (1.66 %). Some of the minor compounds are listed in Table 4 and the gas chromatogram of the *N. muscorum* petroleum ether extract was indicated in Fig. 2a.

**Table 4:** Different bioactive compounds of *N. muscorum* petroleum ether extract analysed using GC-MS.

Peak	Retention Time (RT)	Compounds	Area (%)
1	2.081	Toluene	1.33
2	3.000	Silane, ethenylethoxydiphenyl-	0.89
3	4.501	Cholestan-3ol	1.62
4	4.749	5-Anthracenomethylene)-3-(4-pyridylcarbonyl) rhodanine	1.14
5	5.634	{5-(3,4, 5-trimethoxyphenyl)-1, 3, 4-oxadiazol-2yl}acetic acid	0.31
6	5.697	11-(4-ethoxyphenyl) 2, 3, 5, 7, 11-pentaazatricyclo (7, 4, 0, 0, 2, 6)-trideca-1	0.54
7	5.761	Bycyclo (2, 2, 1) heptanes-1-carboxylic acid	2.14
8	5.850	3-(2-Chloro-4-nitro-phenyl)-2-methyl-3H-quinazolin-4-one	0.69
9	10.487	[1,2,4] Triazole-3-thione, 5-thiophen-2-yl-4-*O*-tolyl-2,4-dihydro	0.66
10	13.165	6-Bromo-4-(2-chloro-phenyl)-quinazoline	0.95
11	13.245	10-Methyl-3, 4, 5, 8, 9, 10-hexahydrooxecin-2-one	2.33
12	14.333	Corynan-17-ol, 18, 19-didehydro-10-methoxy-acetate (ester)	1.86
13	17.533	Xanthine, 1-methyl-7-isopropyl-8-bromo	3.34
14	20.271	9, 10-Secocholesta-5, 7, 10 (19)-triene-3, 24, 25-triol (3-beta, 5Z, 7E)	0.54
15	21.608	Securinan-11-one, 4-methoxy-(4-beta)	0.44
16	25.513	*trans*-1,2-Diethoxycyclohexane	1.79
17	28.686	3-Methyl-4-(phenylthio)-2-prop-2-enyl-2,5-dihydrothiophene 1,1-dioxide	1.21
18	29.170	(2, 2, 6-Trimethyl-bicyclo(4,10)-hept-1-yl)-methanol	0.90
19	32.080	8-methylnonanoic acid	0.34
20	32.114	(Z)-Tritriacont-24-ene-2, 4-dione	0.77
21	32.114	Undecanoic acid, 4,8-dimethyl-10-oxo-methyl ester	1.68
22	32.284	E−8-Methyl-7-dodecen-1-ol acetate	0.77
23	32.914	Oleic acid, 3-(octadecyloxy) propyl ester	1.66
24	35.580	Octadecanoic acid, 16-oxo-, methyl ester	1.68
25	36.292	Bis 2-(ethylhexyl) phthalate	1.53
26	37.333	Methyl 12, 13-tetradecadienoate	1.09
27	39.162	3-Azabutyl-1-ol, 4-cyclopropyl-3,3-dimethyl	0.97
28	39.940	4- heptenal, 6-(acetyloxy)-4-methyl-(E)	1.69
29	41.087	Docosanedoic acid, dimethyl ester	1.66
30	41.636	Undec-10-yonic, decyl ester	0.53
31	43.270	8-Hydroxy-2, 2-dimethyl-8-phenyl-oct-5-en-3-one	1.88
32	43.862	Cucurbitacin, 25-desacetoxy	1.05
33	44.162	Cis Z-11, 12-Epoxytetradecan-1-ol	0.58
34	44.542	Phytol	1.04
35	44.849	3-trsns-(1,1-dimethylethyl)-4-trans-methoxycyclohexanol	0.57

#### 3.2.2 Compounds detected in *N. muscorum* ethanol extract

Similarly, ethanol extract was recorded 10 compounds such as dimethyl sulfone, decane, dodecane, tetradecane, diethyl phthalate, heptadecane, ethyl ester, hexadecanoic acid, diethyl phthalate, ethyl oleate and tetradecanoic acid with a retention time of 4.078, 5.722, 10.983, 16.322, 21.470, 21.712, 24.502, 30.649, 32.445 and 32.628 respectively. The major compounds detected were diethyl phthalate (19.80 %), tetradecane (5.03 %), hexadecanoic acid, ethyl ester (4.10 %) and dodecane (4.03 %) (Table 5). A gas chromatogram of the *N. muscorum* ethanol extract was shown in Fig. 2b.

**Table 5:** Different bioactive compounds of *N. muscorum* ethanol extract analysed using GC-MS.

Peak	Retention Time (RT)	Compounds	Area (%)
1	4.078	Dimethyl sulfone	0.05
2	5.722	Decane	3.01
3	10.983	Dodecane	4.03
4	16.322	Tetradecane	5.03
5	21.470	Diethyl phthalate	19.80
6	21.712	Heptadecane	3.04
7	24.502	Diethyl phthalate	2.05
8	30.649	Hexadecanoic acid, ethyl ester	4.10
9	32.445	Ethyl oleate	3.11
10	32.628	Tetradecanoic acid	2.48

#### 3.2.3 Compounds detected in *N. muscorum* methanol extract

Methanol extract of *N. muscorum* was displayed seven compounds *viz***.,** n-hexadecanoic acid, dimethyl sulfone, hexadecanoic acid, ethyl ester, 6-octadecenoic acid, methyl, diethyl phthalate, 9,12-octadecadienoic acid (Z, Z)-, methyl ester, ester and phytol with a retention time of 4.046, 29.754, 30.222, 30.852, 31.781, 31.861 and 31.975 respectively. The major compounds detected were hexadecanoic acid, ethyl ester (4.02 %), 6-octadecenoic acid, methyl ester (3.04 %) and n-hexadecanoic acid (3.02 %) (Table 6). A gas hromatogram of the *N. muscorum* methanol extract was exhibited in Fig. 2c.

**Table 6:** Different bioactive compounds of *N. muscorum* methanol extract analysed using GC-MS.

Peak	Retention Time (RT)	Compounds	Area (%)
1	4.046	Dimethyl sulfone	0.06
2	29.754	Hexadecanoic acid, ethyl ester	4.02
3	30.222	n- Hexadecanoic acid	3.02
4	30.852	Diethyl phthalate	2.45
5	31.781	9, 12-Octadecadienoic acid (Z,Z)-, methyl ester	2.01
6	31.861	6-Octadecenoic acid, methyl ester	3.04
7	31.975	Phytol	2.04

#### 3.2.4 Compounds detected in S*pirulina* petroleum ether extract

*Spirulina* petroleum ether extract recorded 29 compounds by GC-MS analysis and the compounds were mentioned according to retention time in table number 7. The major compound detected were diethyl phthalate (1.24 %), terephthalic acid, dodecyl 2-ethylhexyl ester octacosane (1.24 %), Bis 2-(ethylhexyl) phthalate (1.18 %), piperidine, 4-propyl (1.16 %), phytol (1.12 %), hexacosane (1.07 %), Phenol, 4-(2-phenylethyl) (1.04 %), stigmastane-3,6-dione (5 alpha) (1.04 %). Some of the minor compounds which are having insecticidal activity are mentioned in Table 7. Though the areas of the compounds were less but the cumulative and synergistic effect of the compounds may caused mortality and affected the various biological parameters of *S. frugiperda*. A gas chromatogram of the *Spirulina* sp. petroleum ether extract was indicated in Fig. 2d.

**Table 7:** Different bioactive compounds of *Spirulina* sp. petroleum ether extract analysed using GC-MS.

Peak	Retention Time (RT)	Compounds	Area (%)
1	2.074	Toluene	0.25
2	4.782	2-*Exo*-hydroxy-5-ketobornane	0.09
3	7.199	Ethyl oleate	0.39
4	7.227	2-Amino-5-bromo-2-fluorobenzophenone	0.45
5	7.747	N-(2-Phenoxyethyl)-4-(2-tetrazol-1yl-ethyl) benzenesulfonamide	0.27
6	11.373	Phenol, 4-(2-phenylethyl)	0.46
7	13.524	Morphinan-6.alpha.-ol, 7, 8-didehydro-3-methoxy-N-methyl	0.41
8	14.089	Benzoic acid, 2, 3, 4, 5-tetrafluoro-6-(2-chlorophenylamino)	0.39
9	17.522	4a.alpha.,4b.beta.-Gibbane-1.alpha.,10.beta.-dicarboxylic acid, 2-beta	0.44
10	18.055	Tyramine, *trans*-trifluoroacetyl	0.33
11	20.195	beta.-*l*-Rhamnofuranoside, 5-*O*-acetyl-thio-octyl	0.41
12	20.433	Octacosane	0.39
13	21.092	Diethyl phthalate	1.24
14	21.125	2, 4, 5-Trichloroaniline, N-trimethylsilyl-	0.10
15	28.216	Hexacosane	1.07
16	31.970	Phytol	1.12
17	33.795	Pentafluoropropionic acid, dodecyl ester	0.13
18	34.580	4 Cyclononen-1-one	0.13
19	35.338	3-Cyclopentylpropionic acid, 2-pentyl ester	0.21
20	35.453	Trimethyl{(2) tetramethyl-1, 3, 2-dioxaborolan-2-yl) ethynyl)}silane	0.47
21	36.286	Bis 2-(ethylhexyl) phthalate	1.18
22	36.633	2-(Ethylenedioxy) ethylamine,N-methyl-N-[4-(1-pymolidinyl-2-butynyl)	0.34
23	37.275	Stigmastane-3, 6-dione (5 alpha)	1.04
24	37.674	2, 3, 16,17-Octadecanetetraone tetraoxime	0.09
25	39.255	Tricosane	0.94
26	39.820	Terephthalic acid, dodecyl 2-ethylhexyl ester	1.24
27	42.086	Hydrazinecarboxamide, 2-cyclohexylidene	0.21
28	43.305	Piperidine, 4-propyl	1.16
29	43.470	Phenol, 2,6-dichloro-4-nitro	1.04

#### 3.2.5 Compounds detected in S*pirulina* ethanol extract

*Spirulina* ethanol extract was recorded eight compounds particularly, dimethyl sulfone, decane, dodecane, tetradecane, diethyl phthalate, hexadecane, diethyl phthalate and hexadecanoic acid, ethyl ester with a retention time of 4.083, 5.719, 10.981, 16.321, 21.466, 21.709, 24.498 and 30.650 respectively and the major compounds detected were diethyl phthalate (19.87 %), dodecane (3.03 %) and hexadecanoic acid, ethyl ester (3.03 %) (Table 8). A gas chromatogram of the *Spirulina* sp. ethanol extract was showed in Fig. 2e.

**Table 8:** Different bioactive compounds of *Spirulina* sp. ethanol extract analysed using GC-MS.

Peak	Retention Time (RT)	Compounds	Area (%)
1	4.083	Dimethyl sulfone	2.04
2	5.719	Decane	1.01
3	10.981	Dodecane	3.03
4	16.321	Tetradecane	2.03
5	21.466	Diethyl phthalate	19.87
6	21.709	Hexadecane	0.18
7	24.498	Diethyl phthalate	2.05
8	30.650	Hexadecanoic acid, ethyl ester	3.03

#### 3.2.6 Compounds detected in *Spirulina* methanol extract

*Spirulina* methanol extract was recorded five compounds namely dimethyl sulfone, octadecanoic acid, methyl ester, phytol, hexadecanoic acid, methyl ester and octadecane with a retention time of 4.057, 29.758, 31.977, 32.257 and 32.646 respectively. Major compounds detected were hexadecanoic acid, methyl ester (4.02 %), octadecane (3.72 %) and octadecanoic acid, methyl ester (3.40 %) (Table 9). The gas chromatogram of the *Spirulina* sp. ethanol extract was represented in Fig. 2f.

**Table 9:** Different bioactive compounds of *Spirulina* sp. methanol extract analysed using GC-MS.

Peak	Retention Time (RT)	Compounds	Area (%)
1	4.057	Dimethyl sulfone	0.05
2	29.758	Hexadecanoic acid, methyl ester	4.02
3	31.977	Phytol	3.02
4	32.257	Octadecanoic acid, methyl ester	3.40
5	32.646	Octadecane	3.72

## 4 Discussion

The toxic effect of *N. muscorum* and *Spirulina* sp. against *S. frugiperda* in the current study is consistent with studies of the other cyanobacteria and algae reported by other authors such as *N. muscorum* and *A. flos aquae* were having more insecticidal activity against second-instar larvae of *A. ipsilon* [16], *N. carneum and Parachorella kessleri* exhibited greater toxicity against, *S. littoralis* [37], *S. platensis* caused 100 percent larval mortality of *S. littoralis* [8] and *S. platensis* and the *Fucus vesiculosus* were considered an effective bio-control agent against broad bean beetle [38]. Since, insecticidal property of petroleum ether extract of green algae, *Scenedesmus acutus* showed greater toxicity against *S. littoralis* [39], ethanol extracts of *N. carneum* and *P. kessleri* exhibited greater mortality of *S. littoralis* [37], *A. flos aquae* ethanol extract caused higher mortality of *S. littoralis* [16], *S. platensis* ethanol extract (7 %) was the most toxic to the larvae of *S. littoralis* [9] and chloroform extracts of green algae, *Bryopsis pennata* exhibited the strongest larvicidal activity followed by *B. pennata* methanol extract against *A. aegypti* [40].

Petroleum ether extract of *N. muscorum* was found to contain 35 compounds during GC-MS analysis of a solvent extract, while petroleum ether extract of *Spirulina* was found to contain 29 compounds. Whereas, the remaining extracts *viz*., ethanol extract of *N. muscorum, Spirulina* reported 10 and 8 compound each. Similarly, methanol extract of *N. muscorum Spirulina* were recorded 7 and 5 compounds respectively. Some similar compounds were present in all the extracts. The compound dimethyl sulfoxide (DMSO) was detected in all the extracts in a higher percent area but, it was not a product of extracted compounds and it was just used as a solvent to dissolve the compounds after extraction, so it was not mentioned from the compounds list detected by GC-MS. The overall study of GC-MS analysis of *Spirulina* sp. and *N. muscorum* extracts recorded many compounds from various extracts; however number of compounds varies with different extracts. It's mainly because each solvents having the capability of dissolving different bioactive compounds [9]. The Soxhlet apparatus was best for extracting more bioactive compounds and this may be due to the boiling liquid directly fall on the material so that the cell wall or tissue ruptures easily and can able to extract all the compounds from the materials [41].

The identified compounds in the present study may have having repellant effect, toxicant and antifeedant or feeding deterrence effect against *S. frugiperda*. The most common compounds present in the extracts were essential oils (phytol, dodecane, decane, heptacosane, hexadecanoic acid etc.), fatty acids (lauric acids, linoleic acid, palmitic acid, oleic acid etc.), antifeedant compounds (5a, Stigmastane-3, 5-diene and cucurbitacin) as well as some of the other repellant and toxicant compounds were responsible for affecting various biological parameters of *S. frugiperda*. Most of the compounds present in the extracts are known to have multiple modes of action, but the detailed modes of action of these compounds are not yet understood. The results of the petroleum ether extracts of *N. muscorum* are in line with the previous reports such as cucurbitacin act as feeding and oviposition deterrence against the mandibulate type of mouthparts [42], naphthalene as essential oil of *Premma angolensis* and *Premma quadrifolia* leaves having repellant activity against *Sitotroga cerealella* [43], naphthalene was harmful to *T. castaneum* and activated genes linked to oxidative stress, metabolism, and reproduction [44]. and brown alga *Sargassum subrepandum* contain heptadecane, heneicosane, nonadecane and 2, 6, 10, 14-tetramethyl-hexadecane having various biological activity [45].

The ethanol extract of *N. muscorum* yields consistent with other studies on the biological activities of *Hypericum perforatum* L. essential oil against *T. castaneum*. The major compounds detected were decane, dodecane, hexadecane and tetradecane were having insecticidal activity particularly fumigant toxicity and antifeedant activity [46], isolated essential oil from *Teucrium polium* L. aerial parts contain decane, dodecane, nonadecane, heptadecane and tridecane were present in the extract having toxicant and antifeedant activity against *T. castenium* [47] and n-hexadecanoic acid and 9–12-octadecadienoic were having potential repellency, toxicity and anti oviposition against whiteflies and mites [48].

The results of methanolic extract of *N. muscorum*, are in confirmation with the previous report such as methanolic extract of *Nostoc* sp. has recorded similar compounds like n-hexadecanoic acid, 9, 12 octadecanoic acid and phytol having an antimicrobial activity [49], methanolic extract of durian, *Durio zibethinus* contains 6-octadecenoic acid, methyl ester and hexadecanoic acid, ethyl ester showed having an antioxidant, antimicrobial and pesticidal activity [50], methanol extract of *Lantana camara* leaf contains many bioactive compounds such as phytol and hexadecanoic acid, methyl ester showed repellant and toxic effects against maize grain weevil [51], At the various doses employed, oleic acid, stearic acid, and linoleic acid demonstrated strong insecticidal action against *Earias insulana*. The three acids' LC50 concentrations extended the duration of the larval and pupal stages, decreased the weight of insect, and altered the overall composition of protein, carbohydrates, and lipids [52], linoleic acid shown a strong toxic effect on *S. littoralis* larvae in their second and fourth instars [53], while Ramos et al. [54] found that linoleic acid exhibited insecticidal properties against *S. frugiperda*.

The results of ethanol and methanol extracts of *Spirulina* sp. are confirmed by the previous studies such as tetradecane is a repellant compound from *Nigella sativa* against *Anopheles gambiae* [55], diethyl phthalate is a potential repellant against the mosquito [56], ethanolic extract of *Acalypha wilkesiana* were contain several compounds like hexadecanoic acid, ethyl ester, n-hexadecanoic acid, 2,6,6-trimethyl, (E)-9-Octadecenoic acid ethyl ester, bicyclo (3.1.1) heptane, octadecanoic acid, 17-methyl-, methyl ester and silane, methyl-exhibited potential insecticidal activity against *C. maculates* [57], methanolic extract of *S. platensis* contains hexadecanoic acid, methyl ester (Palmitic acid, methyl ester), octadecanoic acid, methyl ester (linolenic acid, methyl ester) and phytol in the methanolic extract of *S. platensis* has various biocidal activity [58], methanolic extract of *S. platensis* contains hexadecanoic acid, methyl ester, octadecanoic acid, methyl ester and phytol having antibacterial activity [59]. It indicates these compounds were also present in the cyanobacterial extracts that may affect the growth and development by repelling, antifeedant and toxic effects.

## 5 Conclusion

The extracts of *Nostoc muscorum* and *Spirulina* sp. exhibited insecticidal efficacy against *S. frugiperda*. Though the toxicity of the various extracts differs, each of the cyanobacterial extracts showed insecticidal action against *S. frugiperda* in general. This suggests that different bioactive chemicals can be dissolved by different solvents, leading to a range of outcomes. Petroleum ether extract of *N. muscorum* and *Spirulina* sp. demonstrated notable insecticidal effectiveness among the various treatments, as evidenced by their lowest LC_50_ values and effects on several biological parameters. Through GC-MS analysis, chemicals identified in cyanobacterial extracts were revealed to have potential effects on *S. frugiperda*, including repellent, toxicant, and antifeedant properties as well as feeding deterrent. The most prevalent compounds found in the extracts included fatty acids (lauric acid, linoleic acid, palmitic acid, oleic acid, etc.), essential oils (Phytol, dodecane, decane, heptacosane, hexadecanoic acid, etc.), and antifeedant compounds (5a, Stigmastane-3, 5-diene, and cucurbitacin). A few other repellant and toxicant compounds were also found to have an impact on the biological parameters of *S. frugiperda*. Although the majority of the chemicals found in the extracts are known to have multiple modes of action, their precise mechanisms of action are yet unknown.

## Ethics statement

Not applicable.

## Consent for publication

Not applicable.

## Availability of data and materials

Not applicable.

## Funding

Research supporting project (RSPD2024R741), 10.13039/501100002383King Saud University.

## CRediT authorship contribution statement

**Sharanappa C.H.:** Validation, Methodology, Investigation, Data curation, Writing – original draft. **Bheemanna M:** Writing – review & editing, Visualization, Supervision, Project administration. **Prabhuraj A:** Writing – review & editing, Formal analysis, Validation, Visualization. **Harischandra R. Naik:** Writing – review & editing, Validation, Software, Formal analysis. **Nagaraj M. Naik:** Software, Formal analysis, Data curation, Writing – review & editing. **Saroja N. Rao:** Validation, Software, Data curation, Formal analysis. **Ihab Mohamed Moussa:** Writing – review & editing, Validation, Software. **Roua A. Alsubki:** Validation, Formal analysis, Visualization. **Fazal Ullah:** Writing – review & editing, Supervision, Data curation, Formal analysis. **Hosam O. Elansary:** Software, Resources, Funding acquisition, Formal analysis, Writing – review & editing. **Kariyanna B:** Writing – review & editing, Writing – original draft, Validation, Conceptualization, Formal analysis, Supervision.

## Declaration of competing interest

The authors declare that they have no known competing financial interests or personal relationships that could have appeared to influence the work reported in this paper.
